# Functional heterologous expression of an engineered full length CipA from *Clostridium thermocellum* in *Thermoanaerobacterium saccharolyticum*

**DOI:** 10.1186/1754-6834-6-32

**Published:** 2013-03-01

**Authors:** Devin H Currie, Christopher D Herring, Adam M Guss, Daniel G Olson, David A Hogsett, Lee R Lynd

**Affiliations:** 1 Thayer School of Engineering, Dartmouth College, Hanover, NH 03755, USA; 2 Mascoma Corporation, Lebanon, NH 03766, USA; 3 Oak Ridge National Laboratory, Oak Ridge, TN 37831, USA

**Keywords:** *Thermoanaerobacterium saccharolyticum*, *Clostridium thermocellum*, Cellulosome, Thermophile, Anaerobe, Ethanol, Consolidated bioprocessing

## Abstract

**Background:**

Cellulose is highly recalcitrant and thus requires a specialized suite of enzymes to solubilize it into fermentable sugars. In *C*. *thermocellum*, these extracellular enzymes are present as a highly active multi-component system known as the cellulosome. This study explores the expression of a critical *C*. *thermocellum* cellulosomal component in *T*. *saccharolyticum* as a step toward creating a thermophilic bacterium capable of consolidated bioprocessing by employing heterologously expressed cellulosomes.

**Results:**

We developed an inducible promoter system based on the native *T*. *saccharolyticum xynA* promoter, which was shown to be induced by xylan and xylose. The promoter was used to express the cellulosomal component cipA*, an engineered form of the wild-type *cipA* from *C*. *thermocellum*. Expression and localization to the supernatant were both verified for CipA*. When a Δ*cipA* mutant *C*. *thermocellum* strain was cultured with a CipA*-expressing *T*. *saccharolyticum* strain, hydrolysis and fermentation of 10 grams per liter SigmaCell 101, a highly crystalline cellulose, were observed. This trans-species complementation of a *cipA* deletion demonstrated the ability for CipA* to assemble a functional cellulosome.

**Conclusion:**

This study is the first example of an engineered thermophile heterologously expressing a structural component of a cellulosome. To achieve this goal we developed and tested an inducible promoter for controlled expression in *T*. *saccharolyticum* as well as a synthetic *cipA*. In addition, we demonstrate a high degree of hydrolysis (up to 93%) on microcrystalline cellulose.

## Background

A long sought goal in the cellulosic ethanol field is one-step solubilization and fermentation without added enzymes [1,2]. Such consolidated bioprocessing, or CBP, is considered to be the ultimate low cost approach for cellulose hydrolysis and fermentation [2]. A successful CBP organism must be capable of solubilizing both cellulose and hemicellulose, and also fermenting the resulting sugars to a useful product (e.g., ethanol) at high yield and titer. Unfortunately, no single organism has yet been found or developed that combines these two essential characteristics [3].

Two saccharolytic bacteria of interest for development of CBP-enabling microbes are *Clostridium thermocellum* and *Thermoanaerobacterium saccharolyticum*, both Gram-positive, thermophilic anaerobes. *C*. *thermocellum* exhibits among the highest growth rates on cellulose among described microbes [1], but lacks the ability to ferment hemicellulose. *C*. *thermocellum*’s ability to solubilize crystalline cellulose, as well as other insoluble components of plant biomass, results from its elaborate, multi-protein cellulase complex or cellulosome [4-7]. *T*. *saccharolyticum* readily solubilizes hemicellulose and ferments all common sugars in biomass, but does not solubilize cellulose. This bacterium is highly amenable to genetic manipulation, indeed exhibiting natural competence, and has been engineered to make ethanol at high yields and titers [8-11].

The component of the *C*. *thermocellum* cellulosome with the highest molecular weight is the scaffoldin protein, CipA, which has been implicated in mediating the enzymatic synergy seen in the cellulosome [7,12,13]. The structure of the *C*. *thermocellum* CipA includes one cellulose binding domain or CBD, one type II dockerin which is used to associate with cell wall anchoring proteins, and 9 highly conserved type I cohesins interspaced by flexible linker regions [7]. The type 1 cohesin domains bind with high affinity to type 1 dockerin domains present in over 70 catalytically-active enzymes [14].

Previous studies have largely focused on expressing mini cellulosomes, to date with 4 or fewer cohesin regions, or chimeric “designer” cellulosomes in which cohesin-dockerin pairs from different organisms are used to form complexes with a specified sequence of catalytic proteins [15-31]. Recently a paper reporting *in vitro* assembly of cellulosomes. To date, there have been no reported attempts to engineer thermophiles to heterologously express a cellulosome, although one attempt has been made to express cellulases [32].

In *C*. *thermocellum* the presence or absence of CipA has little effect on activity on phosphoric acid swollen cellulose (PASC), carboxymethyl cellulose (CMC), or β-Glucan, but when absent, results in over an order of magnitude decrease in activity on microcrystalline cellulose [27,33]. With this in mind, microcrystalline cellulose was chosen as a test substrate for cellulosome assembly and complementation.

Heterologous expression of a functional cellulosome system in *T*. *saccharolyticum* is of interest both for fundamentally-oriented studies of microbial cellulose utilization and as a strategy for developing a CBP-enabling microorganism. The logical point of departure for this endeavor is expression of CipA. Here we endeavor to develop an inducible gene expression system in *T*. *saccharolyticum*, synthesize a gene (cipA*) coding for the same amino acid sequence as CipA but with more diversity in the DNA sequences for ease of use, express CipA* in *T*. *saccharolyticum*, and demonstrate functionality by complementing Δ*cipA* mutants of *C*. *thermocellum*.

## Results

### Construction and testing of xylose/xylan inducible promoter system

**Figure 1:**
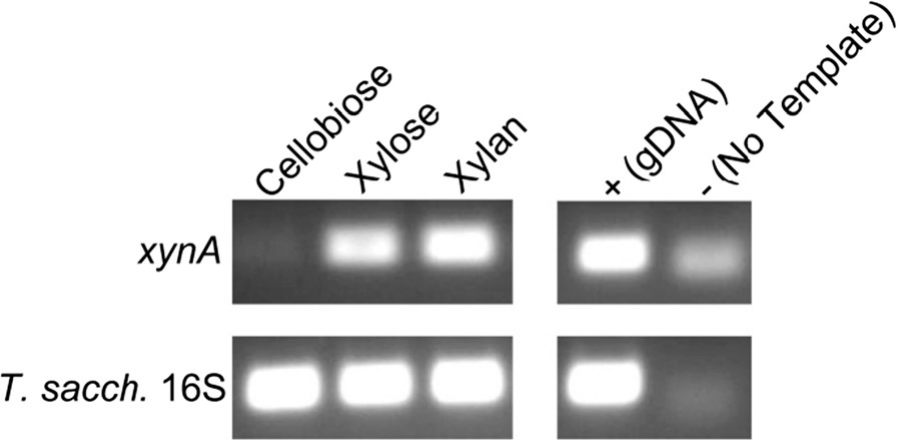
***XynA *induction.** Reverse transcription PCR to determine *xynA* induction during growth on glucose, xylose and xylan, 25 cycles of PCR.

We hypothesized that the promoter and leader sequences of the family 10 endoxylanase *xynA* (Tsac_1459) might be useful in expressing and secreting heterologous proteins in *T*. *saccharolyticum*. To test this hypothesis, we performed RT-PCR to determine gene transcription during growth on cellobiose, xylose, and xylan (Figure 1). The results show that *xynA* is indeed expressed during growth on xylose or xylan and not glucose. In addition it was also observed to be catabolite repressed when glucose and xylose were both present (data not shown). Next, pMC200, which was designed to remove the *xynA* coding region, was transformed into *T*. *saccharolyticum* M1442 to confirm that the regions of homology and resistance cassette were sufficient to drive integration and the removal of the *xynA* open reading frame. *XynA* mutants were tested for growth on xylan and xylose with no substantial defect in growth, in agreement with previous results [34].

**Figure 2:**
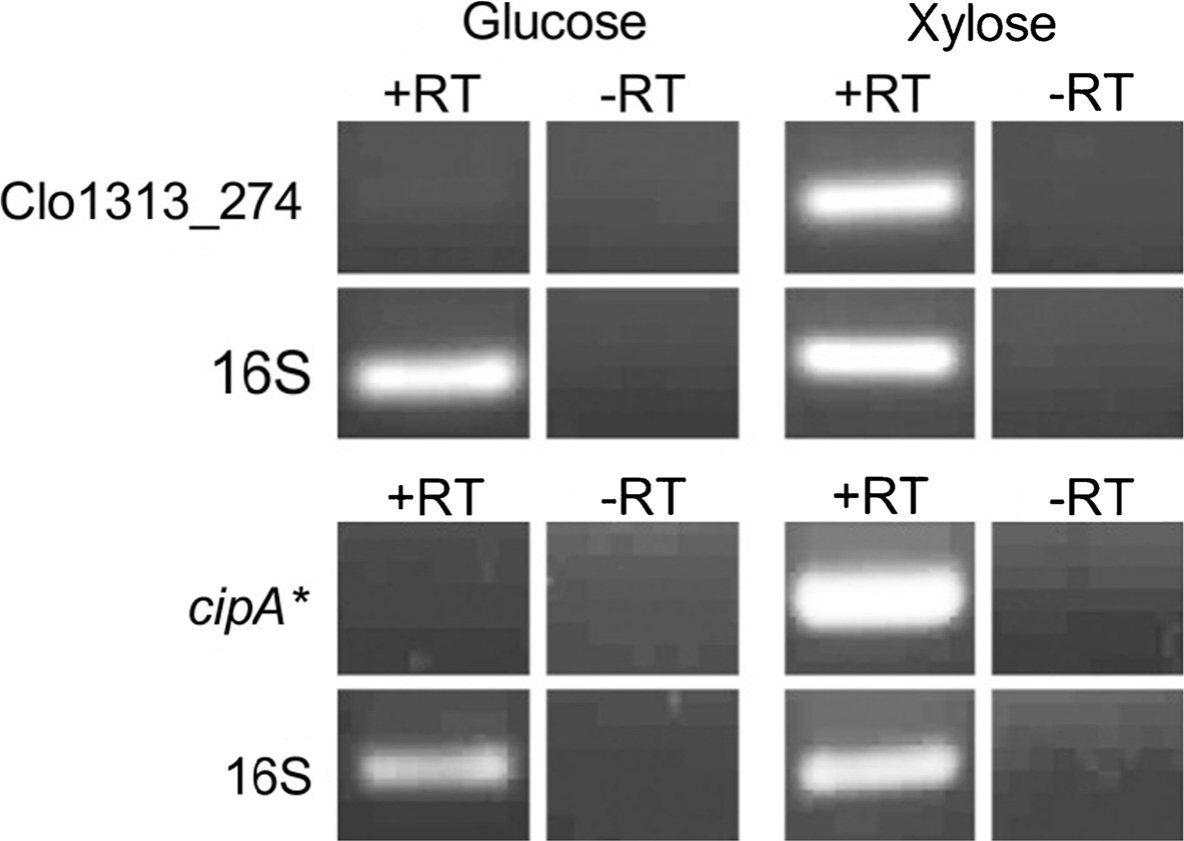
***XynA*’s upstream region as an inducible promoter for use in *T. saccharolyticum*.** Reverse Transcriptase PCR analysis of Clo1313_247 and cipA* under the control of the *xynA* promoter with positive (16S) and negative (no reverse transcriptase added) controls.

A second test of the system was performed by replacing *xynA* with the His tagged coding region for the secreted *C*. *thermocellum* family 48 cellulase *cel48S* (Clo1313_2747), via pMC212. Expression was confirmed via RT-PCR (Figure 2) and was shown to still be regulated by xylose. Unfortunately, the strain did not produce detectable quantities of Cel48S, and as a result no further work was done with the strain. From these data we concluded the *xynA* promoter would function well as an inducible promoter for heterologous gene expression. Furthermore, it allowed cloning of potentially toxic gene products in *E*. *coli*. For example, it was found that when cipA*, discussed in detail below, was placed downstream of the native *T*. *saccharolyticum pta*/*ack* promoter, this resulted in substantial toxic effects to *E*. *coli*. However, no toxicity issues were seen with the *xynA* promoter upstream of cipA*. This indicates that unlike the *pta*/*ack* promoter the regions of the promoter found on pMC200 were insufficient to drive expression in *E*. *coli* in the absence of inducer (xylose).

### Design and synthesis of CipA*

**Figure 3:**
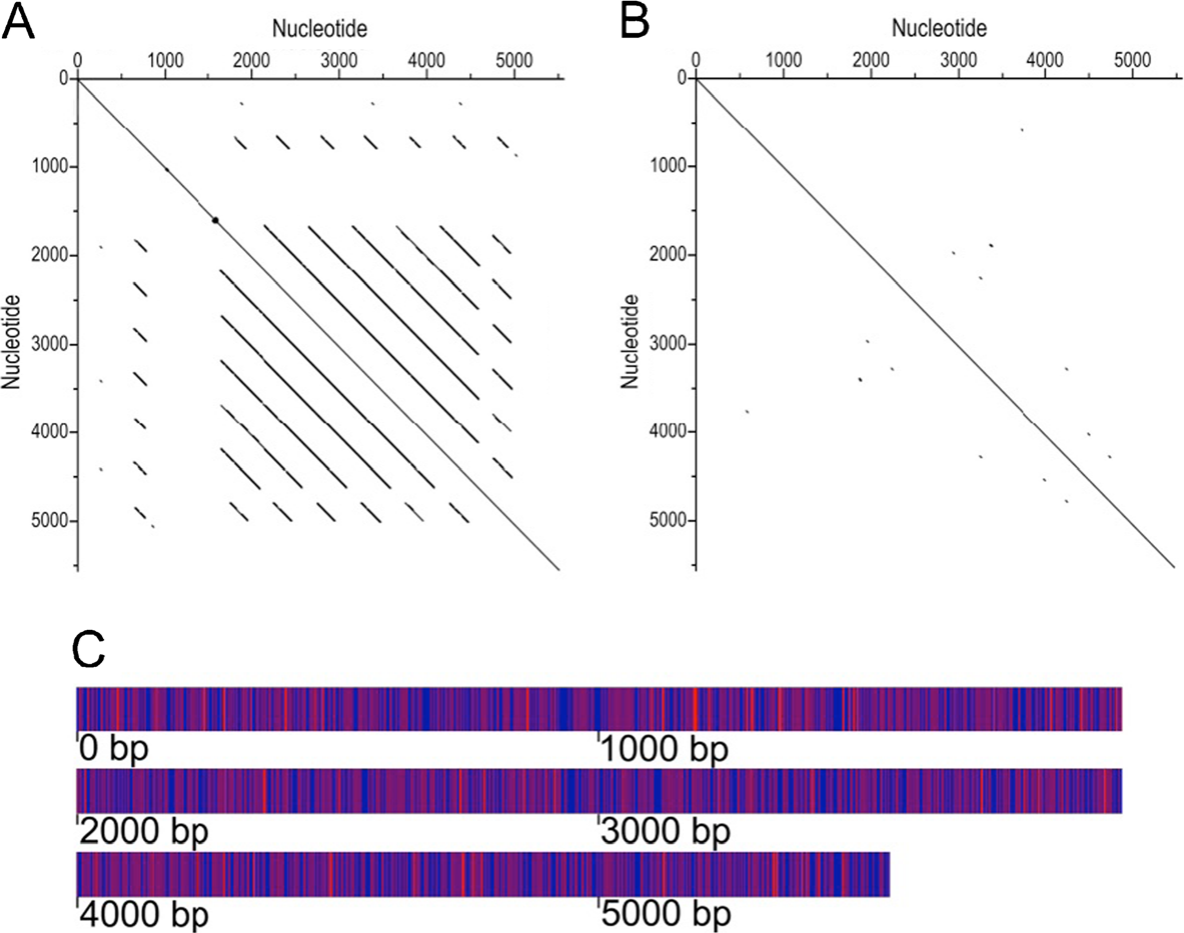
A comparison between the nucleotide sequence of *C. thermocellum’s cipA *and the synthetic cipA*. A) A dot matrix analysis of the wild-type *cipA* with window size of 15 and 0 acceptable mismatches. Positions at which the two sequences are identical at a given position are indicated with a dot. Consecutive identities appear as diagonal lines. **B**) A dot matrix analysis of the engineered cipA* with window size of 15 and 0 acceptable mismatches. **C**) Map of single nucleotide polymorphisms between *cipA* and cipA* with red representing SNPs.

The native *cipA* sequence has large repeated regions likely to be problematic for sequencing as well as genetic stability once introduced into *T*. *saccharolyticum*. To optimize and homogenize *cipA* for expression and sequencing in *T*. *saccharolyticum*, we first identified repeated regions, the largest 4 of these being identical repeats of almost 500 base pairs (Figure 3). To introduce silent mutations to remove this homology we utilized alternate codons with a minimum usage of 15% for a given amino acid in predicted *T*. *saccharolyticum* open reading frames. By manually and iteratively randomizing which alternate codon was used in a given location we were able to break up the repeated regions without generating extended new homologous sequences. It should also be noted that a standard codon optimization would, rather than rectifying the problem, only serve to enhance the redundancy. The result is cipA* which has 1325 nucleotide mismatches to the wild type and no unbroken repeats longer than 19 nucleotides while maintaining a wild type amino acid sequence (Figure 3).

### Expression and localization of CipA*

CipA, like other cellulosomal components, is secreted via the sec pathway which utilizes an N-terminal signal peptide which is cleaved to liberate the mature protein. In order to assure the His tag’s presence in the mature protein, cipA* was tagged at the C-terminus with a 10X His tag via a linker (GGGTGHHHHHHHHHH) for detection via western blot.

A number of methods to concentrate *T*. *saccharolyticum* supernatant were tested including His purification with Ni beads and FPLC. However, the best results were obtained using molecular weight cut off spin columns. Initial attempts included bacterial or mammalian protease inhibitors, but after it became clear that proteolysis was not an issue, their inclusion was discontinued. Xylose was used as an inducer since it was as effective as xylan (Figure 1) but was more practical to use with spin columns. Unlike in *E*. *coli* no negative cellular effects were seen as the result of the presence, or induction, of cipA*. The concentrated protein was washed with 20 mM sodium citrate buffer (pH 5.7) to remove residual sugars to prevent heavy warping of the protein bands during migration on a SDS-PAGE gel.

**Figure 4:**
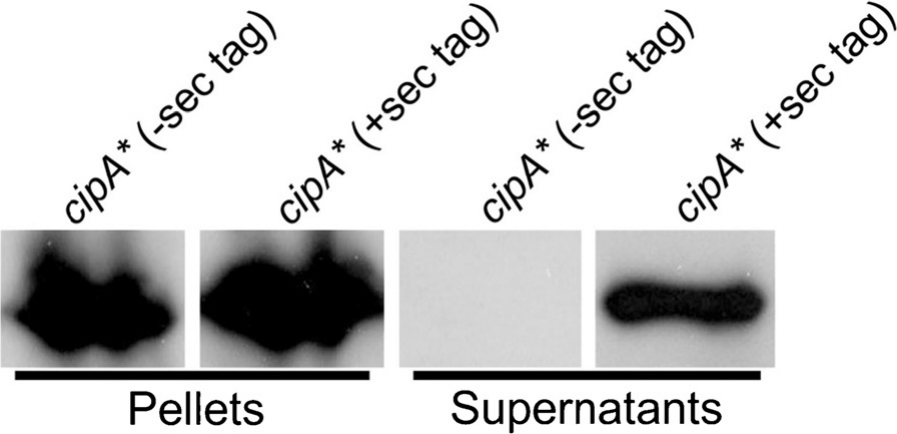
Western blot analysis of the stability and localization of CipA* in *T. saccharolyticum*.

Protein isolated from the supernatant and pellet was subject to western blot analysis, probing for the 10X-His tags. CipA* was observed in both the cytoplasm and in the supernatant under inducing conditions (Figure 4) and was absent when grown on glucose (data not shown). To further confirm active secretion and to rule out the possibility of cell lysis as a means of generating CipA* in the supernatant, a second strain was constructed lacking the predicted native sec tag [35] (Additional file 1: Figure S2) and the localization of cipA* in the two strains was compared. The CipA* derivative lacking a sec tag was only detected in the pellet fraction whereas the CipA* with its sec tag intact was found in both the pellet and the supernatant suggesting lysis was not responsible for presence of CipA* in the supernatant (Figure 4).

Our initial attempts using the standard 100°C denaturation in preparation for running an SDS-PAGE resulted in protein cleavage as others have also observed for *cipA*[36,37]. However, when the denaturation temperature was dropped, or time was decreased, we obtained a single intact band.

### Interspecies complementation

**Figure 5:**
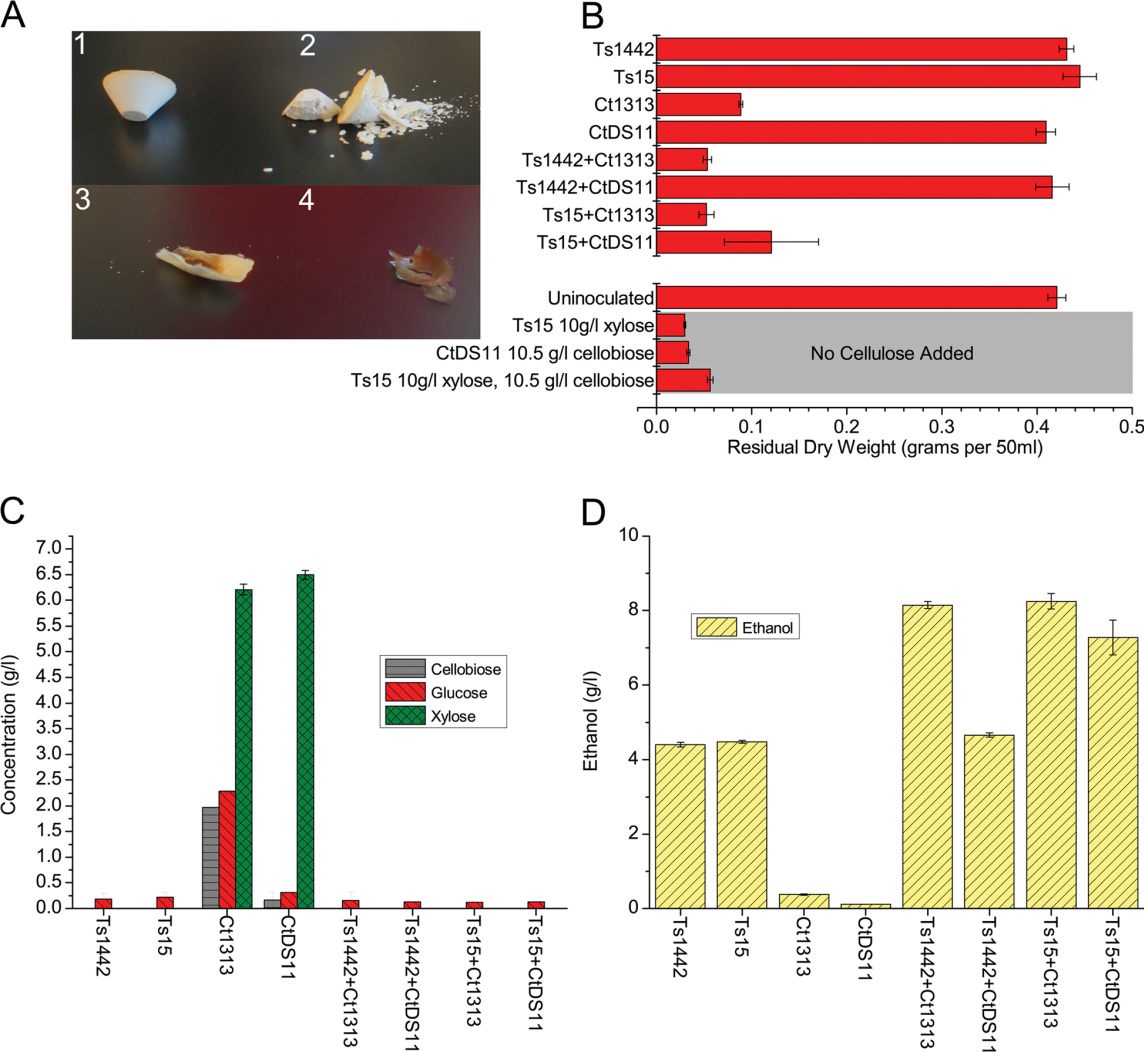
**Results from mono and co-cultures of *T. saccharolyticum *and *C. thermocellum*.** Strain names are given in an abbreviated form for ease of reading. **A**) Representative residual solids with increasing degrees of cellulose solubilization. #1 uninoculated, #2 *T. saccharolyticum* M1442, #3 co-culture between *T. saccharolyticum* DHC15 and *C. thermocellum* DS11, #4 co-culture between wild-type *C. thermocellum* and *T. saccharolyticum* M1442. **B**) The dry weights of residual solids from each of the mono or co-cultures. The three final bars labeled ‘No Cellulose Added’ were not supplied with cellulose and are present to show the maximum contribution made by cell mass alone. Panels **C** and **D** show HPLC data for remaining sugars and ethanol formation, respectively for the parent strain of *T. saccharolyticum* (Ts1442), the CipA* expressing strain of *T. saccharolyticum* (Ts15), the wild type *C. thermocellum* (Ct1313), and the *C. thermocellum cipA* deletion mutant (CtDS11).

Co-cultures of *C*. *thermocellum* and *T*. *saccharolyticum* were used to test the functionality of heterologously expressed CipA*. We utilized a *cipA* deletion strain of *C*. *thermocellum* that shows markedly impaired growth on microcrystalline cellulose but still produces cellulosomal components other than CipA when cultivated on cellobiose. By co-culturing *C*. *thermocellum* Δ*cipA* with the strain of *T*. *saccharolyticum* that expresses CipA*, we hypothesized that CipA*, if functional, would complement the deletion in *C*. *thermocellum* and act as a scaffold thus restoring the ability to hydrolyze cellulose. To evaluate the co-culture’s ability to hydrolyze cellulose we determined residual cellulose/dry weight and product formation (Figure 5).

The wild type *C*. *thermocellum* performed well with respect to cellulose hydrolysis as measured by dry weight. The observed low product formation and unfermented sugars seen with wild type *C*. *thermocellum* are most likely the result of the low starting pH and the lack of pH control resulting in a discontinuation of metabolic, but not enzymatic hydrolysis activity later in growth. No strain of *T*. *saccharolyticum* alone appeared to have any effect on cellulose, but grew entirely on the supplied xylose, nor did the wild type strain of *T*. *saccharolyticum* rescue the cellulose hydrolysis defect in *C*. *thermocellum* strain DS11. Only *T*. *saccharolyticum* expressing cipA* (strain DHC15) was able to restore cellulose hydrolysis functionality to the *C*. *thermocellum* Δ*cipA* (strain DS11).

Finally, we wished to confirm that populated cellulosomes were being formed. Cellulosomes were purified via affinity digestion from co-cultures between DHC15 and DS11 and compared via native PAGE to those from wild type *C*. *thermocellum* and concentrated supernatants from DHC15 and DS11 grown indivigually (Additional file 2: Figure S3). As expected neither DHC15 nor DS11 were able to form cellulosomes when grown independently. When DHC15 and DS11 were grown together cellulosomes were produced with an identical native PAGE migration as those from wild type *C*. *thermocellum*.

These data demonstrate that the *T*. *saccharolyticum*-produced CipA* is capable of gathering and displaying functional cellulosomal enzymes. In addition, the appearance of the pellet further supported the removal of cellulose from the wild type *C*. *thermocellum* and the co-cultures of cipA* and Δ*cipA* as these dry pellets were nearly translucent, suggesting only cell debris, rather than the white cellulose observed in the other samples.

## Discussion

Here we report the expression of an engineered CipA* under the control of a novel inducible promoter in *T*. *saccharolyticum* which allowed for the assembly of active cellulosomes when co-cultured with a *cipA* deletion strain of *C*. *thermocellum*.

The wild-type *cipA* gene has multiple sections with essentially identical DNA sequences, corresponding to the 9 type-I dockerin regions [7]. These repeated sequences can be problematic by complicating sequence verification via routine sequencing technology and could also lead to unwanted partial gene deletion via homologous recombination. Many strains of *E*. *coli* used to heterologously express cellulosomal proteins for biochemical studies are *recA*- [24,27,38]. However, studies such as this one which seek to integrate cellulase genes into the chromosome via native host machinery must use *recA*+ strains thereby exacerbating the challenge of homologous recombination. By using cipA*, designed to avoid repeated sequences, routine sequencing proceeded without difficulty and homologous recombination was not observed.

Toxicity has been observed by other groups working with heterologous expression of *cipA*, and were solved, at least in part, by using inducible promoters in *E*. *coli* and *Lactococcus lactis*[23,27]. For this and other reasons we wished to express cipA* under the control of an inducible promoter. As there have been no inducible promoter systems described for *T*. *saccharolyticum* we designed and tested one based on *xynA*’s promoter. We chose this promoter due to the fact that XynA has been shown to be non-essential [34] and thus could be replaced with a gene of interest. We found that the *xynA* promoter avoided toxicity effects in both *E*. *coli* and *T*. *saccharolyticum*, although apparent toxicity was encountered using the *pta*/*ack* promoter.

In past *in vitro* heterologous cellulosome expression reports significant hydrolysis of microcrystalline cellulose was either not achieved or not tested, with one exception reaching 45% hydrolysis [15,17,18,23,25,28-31]. In the data reported here if we remove the contribution of cell mass to the dry weights and compare that to the uninoculated bottles we see that total cellulose solubilization is between 98 and 100 percent for the co-culture of the CipA* expressing *T*. *saccharolyiticum* and the wild-type *C*. *thermocellum*, and between 71 and 93 percent for the CipA* expressing *T*. *saccharolyiticum* and *C*. *thermocellum* Δ*cipA*. While the trans-complementation co-culture can achieve close to wild type co-culture solubilization in some cases, it is rather variable. The lower hydrolysis from the CipA* expressing *T*. *saccharolyticum* and *C*. *thermocellum* Δ*cipA* co-culture, as compared to that of wild-type *C*. *thermocellum*, may be the result of one or more effects. First, while the cohesin dockerin interaction is quite robust, it is possible that when produced by two different strains the assembly of mature cellulosomes occurs less efficiently than if the components are being secreted simultaneously from the same cell [39,40]. Second, the assembled cellulosomes are incapable of adhering to the surface of the more metabolically active *T*. *saccharolyticum*, and thus are either present free in the media or bound to the surface of *C*. *thermocellum*’s via the native anchor proteins [41-43]. This could result in local product inhibition, and may contribute to the lower than expected hydrolysis [3,44-46].

Krauss *et al*. found that CipA purified from *E*. *coli* was populated with cellulosomal enzymes present in *C*. *thermocellum* supernatants and had near wild-type activity on microcrystalline cellulose [27]. These results led the authors to conclude that the cohesin dockerin interactions are the primary means of cellulosome assembly, an interpretation which our work also supports. Whereas Krauss et al. reported cellulosome assembly *in vitro*, we demonstrate here assembly from components produced by growing cultures with heterologous production using a host that has a temperature optimum compatible with that of the *C*. *thermocellum* cellulosome.

In both Krauss *et al*. and this study, cellulosomes derived from components produced in separate organisms but otherwise unmodified are found to exhibit similar, although somewhat lower, activity on crystalline cellulose as compared to controls with components produced by a single organism. By contrast, studies involving “designer cellulosomes”, in which specific catalytic components bind in a specific order to chimeric scaffoldins, either report several-fold lower activity on crystalline cellulose compared to controls or do not report activity on crystalline cellulose at all. This difference could be because of the importance of a diverse population of cellulosomes with randomly-combined catalytic components, the smaller size of chimeric scaffoldins used in designer cellulosome work, or a combination. The model system reported here, featuring full-sized engineered scaffoldins, is a promising platform for understanding these effects.

An unexpected result of the expression and purification of CipA* in *T*. *saccharolyticum* was the observed instability upon high temperature treatment with SDS-PAGE loading buffer present. While a similar effect has been reported by Morag and Lamed in *C*. *thermocellum*, the conditions used to achieve this result are markedly different [36,37]. In the previous reports, purified protein was subjected to low pH (3.5) or low ionic strength (dialyzed against double distilled water overnight) which resulted in the cleavage of an Asp-Pro peptide bond present in the cohesin domain of CipA. In contrast, no harsh conditions were applied to CipA* from *T*. *saccharolyticum* with supernatant pHs staying above 5.8 and with no dialysis treatment applied. This may indicate a considerable difference between the extracellular environment developed by cultures of *C*. *thermocellum* compared to that of *T*. *saccharolyticum*, and could be important in future attempts at heterologous cellulosome expression.

## Conclusion

Combined with the native ability of *T*. *saccharolyticum* to utilize hemicellulose and the availability of engineered strains that produce ethanol at high yield and titer [8,11], a strain of *T*. *saccharolyticum* with the ability to solubilize cellulose would be a strong candidate organism for CBP. Our results, including expression and secretion of a functional, engineered, full-length CipA, represent a step toward developing such an organism. In addition, we demonstrate a model system in which understanding cellulolytic organisms and their enzyme systems can be tested by systematically reconstructing them.

## Methods

### Microorganisms and growth media

The parent strain for all *T*. *saccharolyticum* strains is M1442 [47], engineered with deletions of the genes for phosphotransacetylase, acetate kinase, and lactate dehydrogenase and expressing genes for urea utilization from *C*. *thermocellum* which serve to buffer acid production [11]. *C*. *thermocellum* DSM1313 was obtained from DSMZ (Deutsche Sammlung von Mikroorganismen und Zellkulturen GmbH, Germany). *C*. *thermocellum* strain DS11, a *cipA* deletion mutant, was generated in our laboratory and is derived from *C*. *thermocellum* DSM1313 Δ*hpt* and was supplied by D. G. Olson [48]. All *T*. *saccharolyticum* strains were grown in modified DSMZ M122 medium [9] with 10 g/l xylose or xylan where noted, 0.5 g/l urea at pH 6.3 and 55°C unless otherwise stated. *C*. *thermocellum* strains were grown in M122 with 10 g/l cellobiose, pH 7.0 at 55°C. All cultures were grown in a Coy anaerobic chamber under a nitrogen, carbon dioxide, and hydrogen gas mix unless otherwise noted.

### Plasmid and strain construction

All plasmids were constructed in *Saccharomyces cerevisiae* FY2 [49] via yeast mediated homologous recombination [50], isolated from yeast with a Zymoprep Yeast Plasmid Miniprep II (Zymo Research, Irvine, CA) and transformed into chemically competent Invitrogen *E*. *coli* TOP10 (Invitrogen Corp, Carlsbad, CA) to generate sufficient quantities of plasmid for transformation into *T*. *saccharolyticum*. Plasmids were then isolated from *E*. *coli* with the QIAGEN Plasmid Mini kit (QIAGEN Inc, Hilden, Germany). *T*. *saccharolyticum* was transformed with plasmid DNA as previously described in a Coy anaerobic chamber [9]. Plasmids and strains are listed in Table 1. Genotypic confirmation for modified strains was obtained via PCR and sequencing. CipA* was synthesized by GeneArt (Additional file 3: Figure S1) [51].

**Table 1:** Strains, plasmids, and primers used in this study

**Strain**	**Description and characteristics**	**Reference**
*T. sacch.* M1442	High titer ethanol producing strain	Lee *et al*., 2011 [47]
*T. sacch.* DHC6	*T. sacch* Δ*xynA*::Clo1313_2747	This study
*T. sacch.* DHC15	*T. sacch* Δ*xynA*::cipA* (wild type sec tag)	This study
*T. sacch.* DHC16	*T. sacch* Δ*xynA*::cipA* (with a deletion in the sec tag)	This study
*C. therm.* DSM 1313	*C. therm* wild type obtained from DSMZ culture collection	DSMZ
*C. therm.* DS11	*C. therm* 1313 Δ*cipA*	Olson *et al.* 2013 [48]
*E. coli* TOP 10	Chemically competent	Invitrogen
*S. cerevisiae* FY2	Uracil Auxotroph used for homologous recombination	Winston *et. al*, 1995 [49]
**Plasmid**	**Description and characteristics**	**Reference**
pMC200	Deletes *xynA* and replaces it with a kanamycin resistance gene and a removable marker	This study
pMC212	Replaces *xynA* with Clo1313_2747, a kanamycin resistance gene, and a removable marker	This study
pMC213	Replaces *xynA* with cipA*, a kanamycin resistance gene, and a removable marker	This study
pMC223	Replaces *xynA* with Δsec tag cipA*, a kanamycin resistance gene, and a removable marker	This study
**Primer**	**Sequence**	**Reference**
xynA_RT_F	TACTTCAGGATGGGTTGGAACAGG	This study
xynA_RT_R	TCCAATTAGCTGTTCTCCCTGTCG	This study
gamA_RT_F	ATATTCACCAGCAACGCTGGCTTC	This study
gamA_RT_R	AATAAGCCTTTGCCAGTTGTCCGC	This study
Clo1313_2747_RT_F	AGTGGCGTTATACAAACGCTCCTG	This study
Clo1313_2747_RT_R	ATACAACGGAAGCAACGGCAGAAC	This study
cipA*_RT_F	ACGACTATCTTTGCCGCTATGATCCC	This study
cipA*_RT_R	ACTTTAGATCCTACGGCAGCAGTGAC	This study
pMC200_up_F	gtctttcgactgagcctttcgttttatttgatgcctggatcttttctggcctttaatggcg	This study
pMC200_up_R	cagctgaagcttcccggggatcctctagagaattcgagctctcttacttcctccctcagtaaatttaatttattg	This study
pMC200_down_F	ctagataggggtcccgagcgcctacgaggaatttgtatcgaaaaaacaaataatctttaagtaaaaaggcagagagg	This study
pMC200_down_R	ccgtcagtagctgaacaggagggacagctgatagaagtcaaatgcgacaaaaaaacgcc	This study

### Reverse transcription PCR

RNA was isolated from cultures of *T*. *saccharolyticum* incubated overnight at 55°C in modified M122 [9] with 10 g/l glucose, xylose, or xylan. RNA was purified with the QIAGEN RNeasy Mini Kit and stored at −80°C. cDNA was generated with the QIAGEN QuantiTect Reverse Transcription Kit. The resulting cDNA was examined for the presence of the transcripts of interest using the primers listed in Table 1.

### Insertion of CipA* into the chromosome

Insertion of cipA* into the chromosome under the control of the *xynA* promoter was achieved via double homologous recombination, selected for by the presence of a kanamycin resistance marker on a nonreplicative plasmid pMC213. The sites of recombination were directly upstream of the start codon and downstream of the stop codon in the *xynA* open reading frame. The size of these regions of homology was 1000 base pairs each, and left the ribosome binding site from *xynA* intact.

### Western blot analysis of his-tagged proteins

*T*. *saccharolyticum* strains were grown to 2/3 maximum OD600. Cells were pelleted, supernatants were filter sterilized and concentrated with Vivaspin 20, PES 10,000 molecular weight cut off centrifugal concentrators (Sartorius Stedim Biotech) as per the manufacturer’s instructions. Samples were washed with two volumes of sodium citrate buffer (20 mM, pH 5.7) at 15°C. Pellets were treated with 20 mg/ml lysozyme in SET buffer (40 mM EDTA, 50 mM Tris–HCl, pH 8.0, 0.75 M sucrose) for 10 minutes to remove the cell wall, pelleted and resuspended in lysis buffer (10 mM Tris pH 7, 0.2% SDS, 1 mM DTT) and incubated at 55°C for 15 minutes. Proteins were denatured at 55°C in loading buffer (5X loading buffer: 6.25 ml 1 M Tris pH 6.8, 2 ml glycerol, 7.3 g SDS, bromophenol blue 0.1%, final pH 6.8). Total protein from either supernatants or lysed cell pellets were analyzed via Western blot with mouse Penta-His (Cat. No. 34660, QIAGEN Inc.) primary, and goat anti-mouse peroxidase conjugate (Cat. No. 31439, Thermo Sci.) secondary antibody. Detection was performed with Western Lightning ECL substrate (PerkinElmer, Waltham, MA) and detected on Kodak X-ray film.

### Co-cultures

50 ml co-cultures were grown in 115 ml nitrogen flushed anaerobic serum bottles agitated in an incubator at 55°C. The medium for the co-cultures was modified DSMZ M122 [9] with 10 g/l xylose and 10 g/l Sigmacell 101 (a microcrystalline cellulose similar to avicel) and 0.5 g/l cellobiose to assist in the initial growth of *C*. *thermocellum*. The initial pH was pH 6.3, previously demonstrated to be suitable for co-cultures between these two organisms [52]. Co-cultures were allowed to grow for 5 days. Cellulosomes were purified via affinity digestion of PASC [27].

### Product formation and dry weight

Dry weight was determined by pelleting the remaining cellulose via centrifugation for 10 minutes at 8000 g, washing twice with deionized water, and drying at 45°C under vacuum for 2 days. Dried pellets were then weighed to determine residual solids. The contribution of cell mass was taken into account with controls containing 10 g/l cellobiose in place of Sigmacell. Residual sugars and ethanol in the supernatants were quantified by HPLC using an Aminex HPX-87H column (Bio-Rad Laboratories, Hercules, CA). Nucleotide sequence accession number.The sequences reported in this paper have been deposited in the GenBank database (accession no. KC675188 [pMC200], KC675189 [pMC212], KC675190 [pMC213], and KC675191 [pMC223]).

## Abbreviations

CBP: Consolidated bioprocessing; CBD: Cellulose binding domain; PASC: Phosphoric acid swollen cellulose; CMC: Carboxymethyl cellulose

## Competing interests

This research was supported in part by Mascoma Corporation, Lebanon NH, with which authors DHC, CDH, DAH and LRL are affiliated. Mascoma Corporation has a commercial interest in the organisms used in this study. The authors DHC and AMG are listed on the international and national pending patents for the sequence of cipA* [52].

## Authors’ contributions

DHC performed the work presented herein and drafted the manuscript. DGO assisted in drafting the manuscript. CDH, AMG, DAH and LRL supervised the work and assisted in drafting the manuscript. All authors read and approved the final manuscript.

## Supplementary Material

Additional file 1 Sequence of cipA* minus the predicted signal peptide with a C-terminal 10X his tag and linker region. Click here for file

Additional file 2 Coomassie stained native PAGE of trans-species formed cellulosomes. Click here for file

Additional file 3 Sequence of cipA* with a C-terminal 10X his tag and linker region. Click here for file
