# Supplementary material for: Functional heterologous expression of an engineered full length CipA from Clostridium thermocellum in Thermoanaerobacterium saccharolyticum
Source: Biotechnol Biofuels. 2013 Mar 1;6:32. doi: 10.1186/1754-6834-6-32 (PMC3598777; doi:10.1186/1754-6834-6-32)
Supplement: Additional file 1 — Sequence of cipA* minus the predicted signal peptide with a C-terminal 10X his tag and linker region. [file 1754-6834-6-32-S1.docx]

>sec_tag_minus_cipA*::10Xhis

atggctactatgacagtagaaatcggaaaggtcactgctgccgtaggatctaaagtagaaatcccgattacattaaagggcgttccgtctaaaggaatggctaattgtgattttgtacttggctatgatccgaatgttcttgaggttactgaggtaaagcctggttctataattaaagatcccgatccaagcaagagttttgactctgcaatttacccagatagaaaaatgattgtttttttattcgctgaagactctggaagaggtacttatgccattacacaagatggggtgtttgcgactatcgttgcgactgtgaagagcgccgctgccgcacccattacattacttgaggtcggggcatttgccgataatgaccttgttgaaatatctacgacttttgttgcaggcggtgttaatcttggcagttctgtgcctacgacgcaacccaatgttccgtctgatggcgttgtcgttgaaataggaaaggtcactgggtctgtcggaacgactgttgaaattccagtatattttagaggcgtcccttcaaagggtatagcaaattgtgactttgtttttaggtatgatccgaatgtattagaaataataggaatcgatccgggagatattatagtggatcctaatccgactaagagttttgacactgctatatatccggatagaaaaattatagtctttcttttcgccgaagatagtggaacaggggcttatgcaattacaaaggatggggtatttgccaagattagggctacggttaagtcttcagccccgggatatatcacttttgatgaggttgggggctttgctgacaatgatttggtggaacagaaggtatcatttattgacggtggggtgaatgtgggaaacgctactccaactaaaggagccactccaacaaatacagctacaccgactaaatctgcaactgcaactccgacaagaccttctgtgccaactaatactcctactaacacaccagcaaacactccagtttcaggaaaccttaaagttgagttttacaattcaaacccttctgatactactaattctatcaatccacaattcaaagtgacaaacactggttcatcagctatcgatttgtcaaaacttactcttaggtattactatacagtggatggtcaaaaggatcaaacattttggtgcgatcacgctgcaatcatcggatctaatggatcttataacggaatcacttcaaatgtgaaagggactttcgtgaagatgagtagtagtacgaacaacgccgacacgtacttagagattagtttcactggcggtacattggagcctggagcccatgtacagattcaggggaggtttgccaagaacgactggagtaactatacacagagtaatgactacagtttcaaaagtgctagtcaattcgttgagtgggaccaggtgactgcgtatttaaacggagtgttagtctggggaaaggagcctggtgggagcgtcgtgccttctacacaaccagttacaacgccgccagctactacaaagccaccggcgacaactaagcctccagccacgacaattccgccatctgatgatcctaatgctatcaagataaaggtcgacactgtcaacgcaaaacctggtgacacggttaacattcccgttaggtttagcggaatacctagcaagggcattgcgaattgcgattttgtttatagttatgacccgaacgttcttgagataattgaaatcaagccgggagaacttatagtggacccgaacccagacaaatctttcgatacagccgtttacccagacagaaaaataatcgtcttcttgtttgcagaggattcaggcactggcgcgtacgcgataacaaaagacggtgtgttcgcaacaatagttgcaaaagtcaaaagtggtgcccccaacgggttaagtgtaataaagttcgttgaagttggcggcttcgccaacaacgatcttgtcgagcagaggacgcagttttttgatggtggcgtaaatgtgggggacactacggtcccaactacaccgacgacacctgtcacgacacctacggacgattcaaacgccgtaaggattaaggttgatactgtgaacgccaaaccgggtgatacggttagaatcccagtgagattcagcggcataccatctaaaggaatcgcgaactgcgatttcgtttactcttatgatccaaacgtgcttgaaattatcgaaatagagcccggagatatcatagtcgatcctaaccccgataaatctttcgatactgctgtgtatccagataggaagatcattgtgtttttgtttgcagaagacagcggcacgggcgcgtacgcaatcacgaaagacggagtgttcgcgacgatcgtcgcaaaggtgaagtcaggagcaccgaatggcttaagtgtcatcaaattcgttgaagttggaggtttcgcaaataatgaccttgtagagcagaaaactcagtttttcgatggtggggtaaacgtaggggacactacggagccagctacgcccacgacgcctgttacaacgcccactacaacggacgatttagacgctgtgaggataaaggttgatacagtgaatgccaaaccaggtgacacagtcaggatcccagtgagattttctggaattccttctaagggaattgctaactgcgacttcgtgtactcatacgacccaaatgtattggagattatagagattgagccgggcgatattatcgtggatccgaaccccgataagtctttcgatacagcggtgtacccggacaggaaaattatagtgtttttgttcgcggaggactcaggtacgggcgcgtatgctattactaaagacggagtattcgctacaatagtagccaaagtcaaatctggtgcccccaacggattgagtgtaatcaagtttgttgaagttggaggatttgcaaacaacgacttagtcgagcaaaaaactcagttttttgacgggggtgttaacgtaggtgatacgacggagcctgcaacacctacaactcccgttactacgccaactactactgacgaccttgacgccgtaagaatcaaagtggatactgttaacgcgaagcctggagatacagttaggatacctgttagattctcagggattccatcaaaaggtatagccaactgtgacttcgtctacagttatgatccaaacgtcttagaaattatcgagatagagcctggtgacataattgtggaccctaacccggacaagagcttcgacacagcggtatatcctgataggaaaataatcgttttccttttcgcagaggattcaggcacaggagcatatgcaataactaaggacggggtgtttgctacgatcgttgcaaaagtgaaggaaggagctcccaacggattaagtgtgattaagttcgtcgaggtcggcgggttcgctaacaatgacttggtagagcagaaaacacagttttttgatggaggagttaatgttggagacacgacggagccagctactccaacaacaccggtcacgactccaacgacaactgacgatttagatgctgtgaggataaaagttgacacagttaacgccaagccaggggacactgtgaggatccctgttaggttcagtgggataccgagtaaggggatagccaattgtgactttgtttacagttatgatcccaacgtattagagataatagaaatcgagcccggagagcttatcgtggaccctaaccccacaaagtcattcgacactgcggtgtacccggataggaaaatgattgtgttcttatttgccgaggatagcggaactggagcatacgcaatcacggaagatggtgtatttgcaactatagttgccaaggtcaagagtggtgctccgaatggacttagtgtaataaaatttgtggaggttggtgggttcgcgaataacgatttagtggagcagaagactcaattcttcgatggaggcgttaacgtcggagacacgactgagcctgccacgccaactacgccagttacaacgccaacaactacggacgacttagacgctgtgagaataaaggttgacacagtcaacgcgaagcctggtgacacggtcaggattccagtcagatttagcgggattcccagtaaaggaattgcaaactgcgactttgtgtatagttacgatccaaacgtcttagagattattgagatagagcctggcgacattatcgtcgaccctaaccctgacaagtcatttgacactgcagtttaccctgacagaaaaattatcgtcttcttattcgcggaggacagcggtacgggtgcgtacgcgatcacgaaagacggcgtttttgcaacaatcgtcgccaaagtcaaagagggggcgccgaacggtttatcagttatcaagttcgtagaggttggcggcttcgcgaataacgatcttgttgaacagaaaacgcaattctttgacggaggtgtcaatgtaggagatacgacggtacccacaacatcacctacaacgacacctcccgagcctacgatcactccgaataaacttacattaaaaataggcagggcggagggaagaccgggagacacagtggaaatccctgtgaatttgtatggtgtcccccagaagggtatcgcctcaggagacttcgttgtatcttacgatccaaacgttttggagattatagaaatagaaccgggcgagttaatagtggatccaaatccaactaaaagtttcgacacagcagtctaccctgacaggaagatgatagtgtttcttttcgccgaggatagcggcacaggggcatatgcaataacggaggatggtgtcttcgccacgatagtggctaaagtgaaggagggagcaccggagggattctctgctattgaaatttctgaatttggagcattcgctgacaacgaccttgtggaggtggagacagacttgatcaacggaggagttcttgttactaataaacctgttattgaaggttataaagtttcaggatatattcttcctgactttagttttgacgccacggtcgcacctcttgtcaaagctggtttcaaggttgagatagtagggacagaactttacgcggtaacggacgcgaatggatacttcgaaatcacaggagttcctgcgaacgccagtggatacacgttgaaaatttctagagctacttaccttgacagggtcatagcgaacgttgttgtgacgggggacacttctgtgagtacgagtcaggctccgatcatgatgtgggttggggacattgtcaaggacaacagtatcaatttattagacgttgcagaggtgattagatgcttcaatgccactaagggtagtgcaaactacgtagaagagttagatatcaacagaaacggagcaataaacatgcaggatatcatgatagttcataagcattttggagctacgtcatctgattacgatgcacaaggaggaggaacaggacatcatcatcatcatcatcatcatcatcattaa

Figure S2. Sequence of *cipA** minus the predicted signal peptide with a C-terminal 10X his tag and linker region.
