# Supplementary material for: Functional heterologous expression of an engineered full length CipA from Clostridium thermocellum in Thermoanaerobacterium saccharolyticum
Source: Biotechnol Biofuels. 2013 Mar 1;6:32. doi: 10.1186/1754-6834-6-32 (PMC3598777; doi:10.1186/1754-6834-6-32)
Supplement: Additional file 2 — Coomassie stained native PAGE of trans-species formed cellulosomes. [file 1754-6834-6-32-S2.docx]

Lane 1 Lane 2 Lane 3 Lane 4


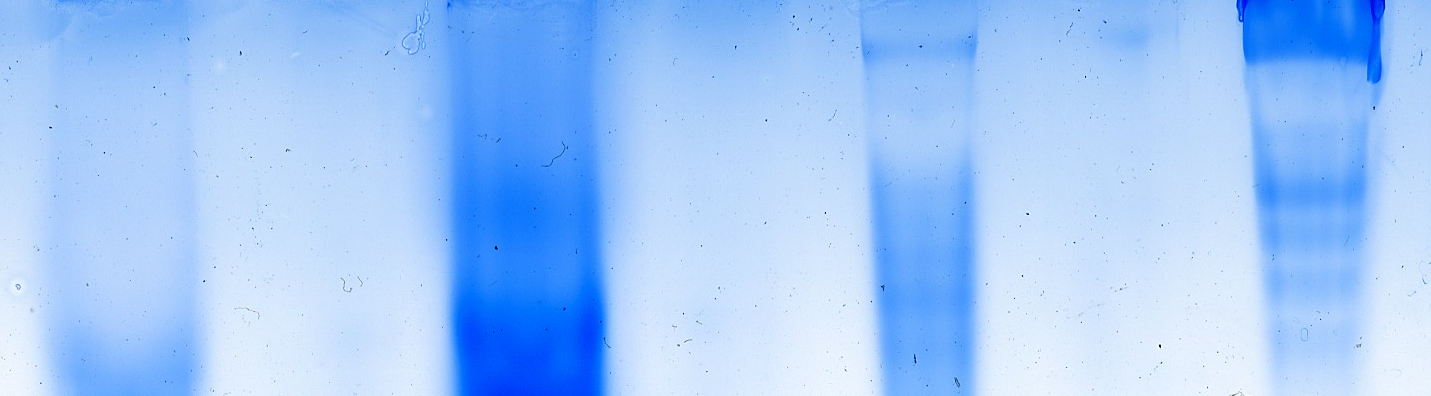


Figure S3. Coomassie stained native PAGE of formed cellulosomes. Lanes 1 and 2 are supernatants from *T. saccharolyticum* DHC15 and *C. thermocellum* DS11 respectively. These were concentrated with 10 kDa molecular weight cut off centrifugal concentrators. Lane 3 contains trans-species formed cellulosomes from co-cultures of *T. saccharolyticum* DHC15 and *C. thermocellum* DS11 isolated via affinity purification with PASC. Lane 4 contains native cellulosomes isolated from cultures of *C. thermocellum* DSM1313 via affinity purification with PASC. Arrows denote formed cellulosomes.
