# Supplementary material for: Functional heterologous expression of an engineered full length CipA from Clostridium thermocellum in Thermoanaerobacterium saccharolyticum
Source: Biotechnol Biofuels. 2013 Mar 1;6:32. doi: 10.1186/1754-6834-6-32 (PMC3598777; doi:10.1186/1754-6834-6-32)
Supplement: Additional file 3 — Sequence of cipA* with a C-terminal 10X his tag and linker region. [file 1754-6834-6-32-S3.docx]

>cipA*::10Xhis

atgaggaaggtgatcagtatgttgttagtggttgctatgttgacgactatctttgccgctatgatccctcaaacggttagtgcagctactatgacagtagaaatcggaaaggtcactgctgccgtaggatctaaagtagaaatcccgattacattaaagggcgttccgtctaaaggaatggctaattgtgattttgtacttggctatgatccgaatgttcttgaggttactgaggtaaagcctggttctataattaaagatcccgatccaagcaagagttttgactctgcaatttacccagatagaaaaatgattgtttttttattcgctgaagactctggaagaggtacttatgccattacacaagatggggtgtttgcgactatcgttgcgactgtgaagagcgccgctgccgcacccattacattacttgaggtcggggcatttgccgataatgaccttgttgaaatatctacgacttttgttgcaggcggtgttaatcttggcagttctgtgcctacgacgcaacccaatgttccgtctgatggcgttgtcgttgaaataggaaaggtcactgggtctgtcggaacgactgttgaaattccagtatattttagaggcgtcccttcaaagggtatagcaaattgtgactttgtttttaggtatgatccgaatgtattagaaataataggaatcgatccgggagatattatagtggatcctaatccgactaagagttttgacactgctatatatccggatagaaaaattatagtctttcttttcgccgaagatagtggaacaggggcttatgcaattacaaaggatggggtatttgccaagattagggctacggttaagtcttcagccccgggatatatcacttttgatgaggttgggggctttgctgacaatgatttggtggaacagaaggtatcatttattgacggtggggtgaatgtgggaaacgctactccaactaaaggagccactccaacaaatacagctacaccgactaaatctgcaactgcaactccgacaagaccttctgtgccaactaatactcctactaacacaccagcaaacactccagtttcaggaaaccttaaagttgagttttacaattcaaacccttctgatactactaattctatcaatccacaattcaaagtgacaaacactggttcatcagctatcgatttgtcaaaacttactcttaggtattactatacagtggatggtcaaaaggatcaaacattttggtgcgatcacgctgcaatcatcggatctaatggatcttataacggaatcacttcaaatgtgaaagggactttcgtgaagatgagtagtagtacgaacaacgccgacacgtacttagagattagtttcactggcggtacattggagcctggagcccatgtacagattcaggggaggtttgccaagaacgactggagtaactatacacagagtaatgactacagtttcaaaagtgctagtcaattcgttgagtgggaccaggtgactgcgtatttaaacggagtgttagtctggggaaaggagcctggtgggagcgtcgtgccttctacacaaccagttacaacgccgccagctactacaaagccaccggcgacaactaagcctccagccacgacaattccgccatctgatgatcctaatgctatcaagataaaggtcgacactgtcaacgcaaaacctggtgacacggttaacattcccgttaggtttagcggaatacctagcaagggcattgcgaattgcgattttgtttatagttatgacccgaacgttcttgagataattgaaatcaagccgggagaacttatagtggacccgaacccagacaaatctttcgatacagccgtttacccagacagaaaaataatcgtcttcttgtttgcagaggattcaggcactggcgcgtacgcgataacaaaagacggtgtgttcgcaacaatagttgcaaaagtcaaaagtggtgcccccaacgggttaagtgtaataaagttcgttgaagttggcggcttcgccaacaacgatcttgtcgagcagaggacgcagttttttgatggtggcgtaaatgtgggggacactacggtcccaactacaccgacgacacctgtcacgacacctacggacgattcaaacgccgtaaggattaaggttgatactgtgaacgccaaaccgggtgatacggttagaatcccagtgagattcagcggcataccatctaaaggaatcgcgaactgcgatttcgtttactcttatgatccaaacgtgcttgaaattatcgaaatagagcccggagatatcatagtcgatcctaaccccgataaatctttcgatactgctgtgtatccagataggaagatcattgtgtttttgtttgcagaagacagcggcacgggcgcgtacgcaatcacgaaagacggagtgttcgcgacgatcgtcgcaaaggtgaagtcaggagcaccgaatggcttaagtgtcatcaaattcgttgaagttggaggtttcgcaaataatgaccttgtagagcagaaaactcagtttttcgatggtggggtaaacgtaggggacactacggagccagctacgcccacgacgcctgttacaacgcccactacaacggacgatttagacgctgtgaggataaaggttgatacagtgaatgccaaaccaggtgacacagtcaggatcccagtgagattttctggaattccttctaagggaattgctaactgcgacttcgtgtactcatacgacccaaatgtattggagattatagagattgagccgggcgatattatcgtggatccgaaccccgataagtctttcgatacagcggtgtacccggacaggaaaattatagtgtttttgttcgcggaggactcaggtacgggcgcgtatgctattactaaagacggagtattcgctacaatagtagccaaagtcaaatctggtgcccccaacggattgagtgtaatcaagtttgttgaagttggaggatttgcaaacaacgacttagtcgagcaaaaaactcagttttttgacgggggtgttaacgtaggtgatacgacggagcctgcaacacctacaactcccgttactacgccaactactactgacgaccttgacgccgtaagaatcaaagtggatactgttaacgcgaagcctggagatacagttaggatacctgttagattctcagggattccatcaaaaggtatagccaactgtgacttcgtctacagttatgatccaaacgtcttagaaattatcgagatagagcctggtgacataattgtggaccctaacccggacaagagcttcgacacagcggtatatcctgataggaaaataatcgttttccttttcgcagaggattcaggcacaggagcatatgcaataactaaggacggggtgtttgctacgatcgttgcaaaagtgaaggaaggagctcccaacggattaagtgtgattaagttcgtcgaggtcggcgggttcgctaacaatgacttggtagagcagaaaacacagttttttgatggaggagttaatgttggagacacgacggagccagctactccaacaacaccggtcacgactccaacgacaactgacgatttagatgctgtgaggataaaagttgacacagttaacgccaagccaggggacactgtgaggatccctgttaggttcagtgggataccgagtaaggggatagccaattgtgactttgtttacagttatgatcccaacgtattagagataatagaaatcgagcccggagagcttatcgtggaccctaaccccacaaagtcattcgacactgcggtgtacccggataggaaaatgattgtgttcttatttgccgaggatagcggaactggagcatacgcaatcacggaagatggtgtatttgcaactatagttgccaaggtcaagagtggtgctccgaatggacttagtgtaataaaatttgtggaggttggtgggttcgcgaataacgatttagtggagcagaagactcaattcttcgatggaggcgttaacgtcggagacacgactgagcctgccacgccaactacgccagttacaacgccaacaactacggacgacttagacgctgtgagaataaaggttgacacagtcaacgcgaagcctggtgacacggtcaggattccagtcagatttagcgggattcccagtaaaggaattgcaaactgcgactttgtgtatagttacgatccaaacgtcttagagattattgagatagagcctggcgacattatcgtcgaccctaaccctgacaagtcatttgacactgcagtttaccctgacagaaaaattatcgtcttcttattcgcggaggacagcggtacgggtgcgtacgcgatcacgaaagacggcgtttttgcaacaatcgtcgccaaagtcaaagagggggcgccgaacggtttatcagttatcaagttcgtagaggttggcggcttcgcgaataacgatcttgttgaacagaaaacgcaattctttgacggaggtgtcaatgtaggagatacgacggtacccacaacatcacctacaacgacacctcccgagcctacgatcactccgaataaacttacattaaaaataggcagggcggagggaagaccgggagacacagtggaaatccctgtgaatttgtatggtgtcccccagaagggtatcgcctcaggagacttcgttgtatcttacgatccaaacgttttggagattatagaaatagaaccgggcgagttaatagtggatccaaatccaactaaaagtttcgacacagcagtctaccctgacaggaagatgatagtgtttcttttcgccgaggatagcggcacaggggcatatgcaataacggaggatggtgtcttcgccacgatagtggctaaagtgaaggagggagcaccggagggattctctgctattgaaatttctgaatttggagcattcgctgacaacgaccttgtggaggtggagacagacttgatcaacggaggagttcttgttactaataaacctgttattgaaggttataaagtttcaggatatattcttcctgactttagttttgacgccacggtcgcacctcttgtcaaagctggtttcaaggttgagatagtagggacagaactttacgcggtaacggacgcgaatggatacttcgaaatcacaggagttcctgcgaacgccagtggatacacgttgaaaatttctagagctacttaccttgacagggtcatagcgaacgttgttgtgacgggggacacttctgtgagtacgagtcaggctccgatcatgatgtgggttggggacattgtcaaggacaacagtatcaatttattagacgttgcagaggtgattagatgcttcaatgccactaagggtagtgcaaactacgtagaagagttagatatcaacagaaacggagcaataaacatgcaggatatcatgatagttcataagcattttggagctacgtcatctgattacgatgcacaaggaggaggaacaggacatcatcatcatcatcatcatcatcatcattaa

Figure S1. Sequence of *cipA** with a C-terminal 10X his tag and linker region.
